# Foot-and-mouth disease virus infection inhibits LGP2 protein expression to exaggerate inflammatory response and promote viral replication

**DOI:** 10.1038/cddis.2017.170

**Published:** 2017-04-13

**Authors:** Zixiang Zhu, Chuntian Li, Xiaoli Du, Guoqing Wang, Weijun Cao, Fan Yang, Huanhuan Feng, Xiangle Zhang, Zhengwang Shi, Huanan Liu, Hong Tian, Dan Li, Keshan Zhang, Xiangtao Liu, Haixue Zheng

**Affiliations:** 1 State Key Laboratory of Veterinary Etiological Biology, National Foot and Mouth Diseases Reference Laboratory, Key Laboratory of Animal Virology of Ministry of Agriculture, Lanzhou Veterinary Research Institute, Chinese Academy of Agricultural Sciences, Lanzhou, China

## Abstract

The role of the innate immune protein LGP2 (laboratory of genetics and physiology 2) in FMDV-infected cells remains unknown. Here, we demonstrate the antiviral role of LGP2 during FMDV infection. FMDV infection triggered LGP2 mRNA expression but reduced protein expression. Overexpression of LGP2 suppressed FMDV replication, and the inflammatory response was significantly inhibited by LGP2 in virus-infected cells. The N-terminal DExDc and the C-terminal regulatory domain regions of LGP2 were essential for LGP2-mediated antiviral activity against FMDV. Disruption of RNA recognition by LGP2 is suggested to abolish completely LGP2-mediated antiviral activity against FMDV. FMDV leader protein (L^pro^), as well as the 3C^pro^ and 2B proteins were determined to possess the ability to induce reduction of LGP2 protein expression. 2B-induced reduction of LGP2 was independent of cleavage of eukaryotic translation initiation factor 4 gamma; and the proteasomes, lysosomes or caspase-dependent pathways were not involved in this process. The C-terminal amino acids of 101–154 were essential for 2B-induced reduction of LGP2 and upregulation of inflammatory response. Direct interaction was demonstrated between LGP2 and 2B. Our results describe the antiviral role of LGP2 against FMDV and a novel antagonistic mechanism of FMDV that is mediated by 2B protein.

Foot-and-mouth disease is an acute and highly contagious disease of cloven-hooved animals, and particularly affects pigs and cattle. Foot-and-mouth disease virus (FMDV) is a single-stranded positive-sense RNA virus that belongs to the genus *Aphthovirus* within the family *Picornaviridae*.^1^ The viral RNA genome is ~8.5 kb long and encodes a polyprotein. The polyprotein is subsequently processed, and generates four structural proteins, VP1, VP2, VP3 and VP4, and eight non-structural proteins, leader protein (L^pro^), 2A, 2B, 2C, 3A, 3B, 3C^pro^ and 3D^pol^.^2^ It has been suggested that different viral proteins counteract innate immune responses through many different mechanisms. For example, VP3 protein inhibits the expression of virus-induced signalling adapter to inhibit the interferon (IFN) signalling pathway.^3^ L^pro^ induces cleavage of various antiviral host proteins to suppress antiviral responses.^4, 5, 6^ 3C^pro^ cleaves eukaryotic translation initiation factors eIF4A and eIF4G, the nuclear factor kappa B (NF-*κ*B) essential modulator and karyopherin *α*1 to abate innate immune signalling, and blocks nuclear translocation of signal transducer and activator of transcription (STAT)1/STAT2 to antagonize the IFN signalling pathway.^7, 8, 9, 10^

The activated innate immune system results in the expression of various antiviral proteins and subsequently induces a series of antiviral responses to suppress viral replication.^11^ Pattern-recognition receptors (PRRs) are responsible for pathogen recognition to initiate and modulate antiviral innate immune responses.^12, 13^ Pattern-recognition receptors include Toll-like receptors, retinoic acid-inducible gene (RIG)-I-like receptors (RLRs), NOD-like receptors and C-type lectin receptors. These pattern-recognition receptors trigger intracellular signalling cascades by different pathways.^12^ RLRs are a family of DExD/H box RNA helicases containing RIG-I, melanoma differentiation-associated protein (MDA)-5 and LGP2 (laboratory of genetics and physiology 2); all of which include a DExD/H box helicase domain.^14^ However, the three RLRs can show different roles during various viral infections.^15^ LGP2 is a homologue of RIG-I and MDA5, but lacks the caspase activation and recruitment domain and is currently thought to function differentially with RIG-I- and MDA5-mediated functions.^16, 17^

LGP2 reveals disparate biological activities during different virus infections.^16, 18^ Overexpression of LGP2 suppresses Newcastle disease virus and Sendai virus signalling to the type I IFN pathway.^14, 19^ LGP2 negatively regulates IFN induction through inhibition of RIG-I function by both RNA-dependent and -independent mechanisms.^19, 20^ In contrast to the negative regulatory role in RIG-I-mediated signalling, it has conversely been shown that LGP2 performs a positive regulatory and synergistic role in MDA5-dependent signalling,^21^ thus showing antiviral activity against encephalomyocarditis virus (EMCV) and poliovirus.^22^ EMCV and poliovirus are picornaviruses that are thought to be mainly sensed by MDA5 rather than RIG-I.^23, 24^ In LGP2 knockout cells, EMCV-induced type I IFN signalling is significantly impaired, and LGP2 knockout mice are highly susceptible to EMCV and poliovirus infection.^22, 24^

Different roles of RLRs have been revealed among different picornavirus infections.^16, 23^ In this study, we investigated the state and antiviral effect of LGP2 during FMDV infection. FMDV L^pro^, 3C^pro^ and 2B proteins were suggested to perform antagonistic roles to inhibit LGP2-mediated antiviral activity.

## Results

### FMDV infection triggered LGP2 transcription and reduced LGP2 protein levels

**Figure 1:**
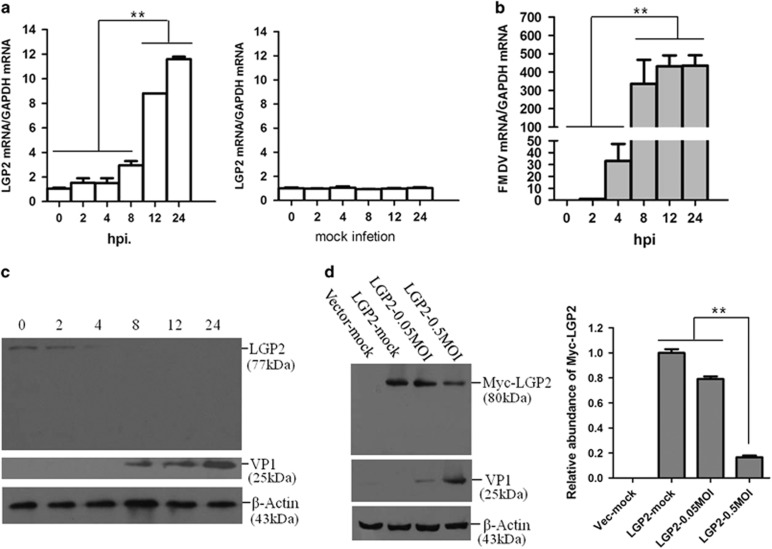
FMDV infection triggers LGP2 mRNA and decreases LGP2 protein expression levels. (**a, b**) PK-15 cells were mock-infected or infected with FMDV (0.5 MOI) for 0, 2, 4, 8, 12 or 24 h, and LGP2 mRNA and viral RNA were detected by qPCR. (**c**) PK-15 cells were infected with FMDV (0.5 MOI) for 0, 2, 4, 8, 12 or 24 h, and endogenous LGP2 and viral VP1 proteins were detected by western blotting. (**d**) PK-15 cells were transfected with Myc-vector (2 μg) or Myc-LGP2 plasmids (2 μg), and Myc-LGP2-transfected cells were mock-infected or infected with 0.05 or 0.5 MOI of FMDV at 12 hpt. Expression of Myc-LGP2 and viral VP1 proteins was detected by western blotting at 12 hpi. The change in abundance of Myc-LGP2 in the transfectants was analysed by densitometric analysis using ImageJ Software and normalized to *β*-actin (right panel).

To explore the potential role of LGP2 during FMDV infection, we first investigated the state of LGP2 in FMDV-infected cells. PK-15 cells were infected by FMDV at an MOI of 0.5, and the dynamics of LGP2 were determined. Transcripts of LGP2 were found to be significantly upregulated at 8 hpi and gradually increased as the infection progressed (Figure 1a). No marked changes in LGP2 mRNA were detected in mock-infected cells. Viral RNA was also detected to exploit the correlation between LGP2 expression and virus replication, which indicated that the viral RNA significantly increased at 4 hpi, and gradually increased, similar to LGP2 (Figure 1b). We also detected the protein abundance of LGP2 at the indicated time points; however, LGP2 is present at low levels in the uninfected cell as previously suggested.^25^ FMDV infection resulted in a gradual decrease of LGP2 protein levels as infection progressed and no cleaved bands were observed by western blotting detection (Figure 1c). FMDV-induced decrease of LGP2 was confirmed by detection of the protein levels of the Myc-LGP2-transfected cells that were mock-infected or infected by FMDV. It also demonstrated that LGP2 protein expression was significantly reduced in the FMDV-infected cells in comparison with the mock-infected cells (Figure 1d).

### Overexpression of LGP2 inhibited FMDV replication

**Figure 2:**
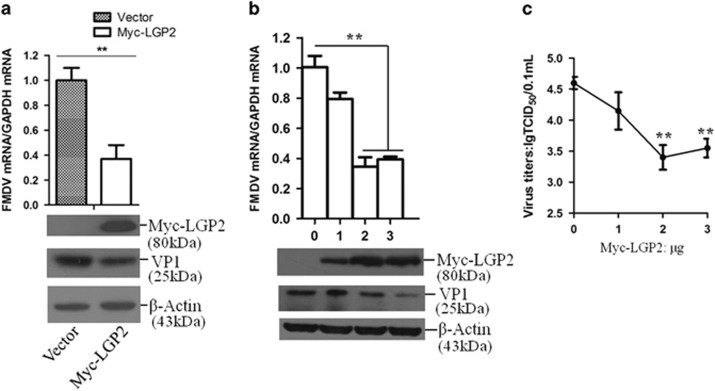
Overexpression of LGP2 suppresses FMDV replication. (**a**) PK-15 cells were transfected with Myc-vector (2 μg) or Myc-LGP2 plasmids (2 μg), and the transfected cells were infected with FMDV (0.5 MOI) at 24 hpt. Expression of Myc-LGP2 and viral VP1 proteins was detected by western blotting, and viral RNA was detected by qPCR, at 12 hpi. (**b**) PK-15 cells were transfected with increasing amounts of Myc-LGP2 plasmids (0, 1, 2 or 3 μg) and the Myc-vector was used in the transfection process to ensure that the cells received the same amounts of total DNA plasmids. The transfected cells were infected with FMDV (0.5 MOI) at 24 hpt. Expression of Myc-LGP2 and viral VP1 proteins was detected by western blotting, and viral RNA was detected by qPCR, at 12 hpi; (**c**) and the viral titres were detected by TCID_50_ assay. All the experiments were repeated three times with similar results.

To explore whether LGP2 had an inhibitory role in FMDV infection, we transiently overexpressed LGP2 in PK-15 cells, and the cells were infected with FMDV at 24 h post-transfection (hpt). The infected cells were harvested at 12 hpi for analysis of viral replication. Expression of Myc-LGP2 was confirmed by western blot analysis; it indicated that both the viral RNA and viral protein levels were decreased in LGP2 overexpressing cells (Figure 2a). A dose-dependence assay was performed, and the viral mRNA and protein levels were used as indicators of viral replication. The increasing amounts of Myc-LGP2 revealed an increased inhibitory effect on FMDV replication, in a dose-dependent manner (Figure 2b). The viral titres were also detected to confirm the antiviral role of LGP2, which revealed a similar effect to that detected by western blotting analysis (Figure 2c).

### Overexpression of LGP2 regulated expression of various cytokines during FMDV infection

**Figure 3:**
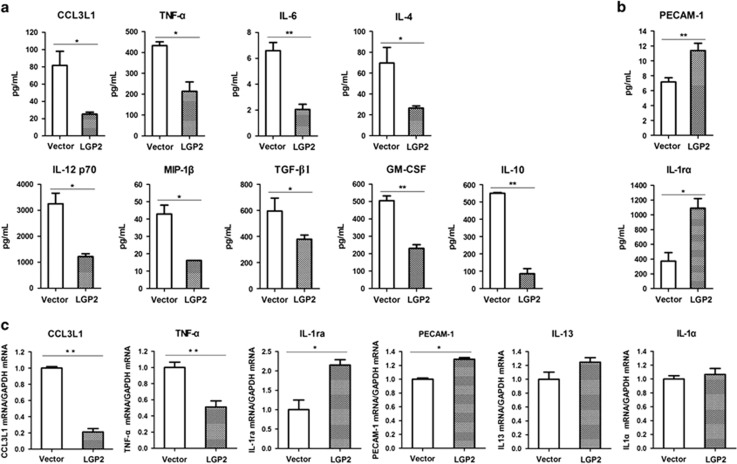
LGP2 suppresses several cytokines expression during FMDV infection. PK-15 cells were transfected with Myc-vector or Myc-LGP2 plasmids, and transfected cells were infected with FMDV at 24 hpt. Cytokine expression levels in the cell lysates was detected at 18 hpi using porcine cytokine array kits (RayBiotech). (**a**) Downregulation of cytokines was greater in LGP2 overexpressing cells than vector-transfected cells after FMDV infection. (**b**) Upregulation of cytokines was greater in LGP2-overexpressing than vector-transfected cells after FMDV infection. (**c**) qPCR analysis of mRNA abundance of six determined cytokines.

In the transgenic mouse overexpressing LGP2, influenza A virus (IAV)-triggered detrimental inflammatory response was significantly downregulated comparing with that in the wild-type mouse infected by the virus.^26^ Therefore, we explored the inflammatory response in LGP2-overexpressed and vector-transfected cells that were infected by FMDV. The antibody arrays against 20 porcine cytokines were used according to the manufacturer's protocol. The expression of the 20 cytokines in the virus-infected cells were detected and analysed. It showed that expression of CCL3L1, TNF-*α*, IL-6, IL-4, IL-12, MIP-1*β*, TGF-*β*1, GM-CSF and IL10 were significantly lower in LGP2-overexpressing cells than in vector-transfected cells after FMDV infection (Figure 3a). Expression of IL1R*α* and PECAM-1 was significantly enhanced (Figure 3b). However, the expression of the other nine cytokines was not significantly affected by upregulation of LGP2 (Supplementary Figure 1). Expression of CCL3L1, TNF-*α*, IL-1R*α*, PECAM-1, IL-13 and IL-1*α* was further detected using qPCR to confirm the results of the antibody arrays. The mRNA expression levels also showed similar results to the antibody array results (Figure 3c). These results suggested that LGP2 suppressed the inflammatory response in FMDV-infected cells.

### Knockdown or knockout of LGP2 promoted FMDV replication

**Figure 4:**
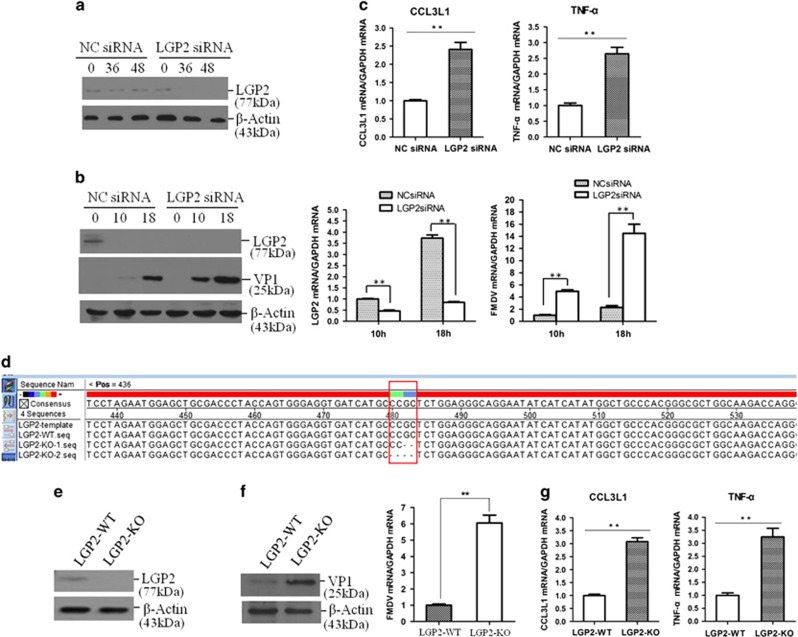
Loss of LGP2 promotes inflammatory response and FMDV replication. (**a**) PK-15 cells were transfected with NC siRNA or LGP2 siRNA, and expression of endogenous LGP2 protein was detected at 0, 36 and 48 hpt. (**b**) PK-15 cells were transfected with NC siRNA or LGP2 siRNA for 36 h, followed by infection with equal amounts of FMDV (0.5 MOI) for 0, 10 and 18 h. Expression of endogenous LGP2 and viral proteins was detected by western blotting. Expression of LGP2 mRNA and viral RNA was detected by qPCR. (**c**) Expression of CCL3L1 and TNF-*α* mRNA in the NC siRNA- and LGP2 siRNA-transfected cells infected by FMDV for 18 h was detected by qPCR. (**d**) Alignment of the LGP2 genomic reference sequence, the LGP2-WT, LGP2-KO-1 and LGP2-KO-2 DNA sequences using LaserGene software. The red box indicates the regions that were mutated. (**e**) Confirmation of successful knockout of LGP2 in the LGP2-KO cell line by western blotting. (**f**) Equal amounts of LGP2-WT and LGP2-KO cells were infected by FMDV (0.5 MOI) for 10 h, and viral protein (left panel) and RNA (right panel) were detected. (**g**) Expression of CCL3L1 and TNF-*α* mRNA were determined.

The RNA interference was used to downregulate LGP2 expression and confirm the antiviral role of LGP2. Expression of LGP2 was significantly downregulated by transfection with LGP2 siRNA, which confirmed the efficiency of LGP2 siRNA (Figure 4a). The FMDV replication status in the NC siRNA- or LGP2 siRNA-transfected cells was analysed and compared at 10 and 18 hpi. It revealed that the viral RNA and viral protein levels were higher in LGP2 siRNA cells (Figure 4b), indicating that downregulation of LGP2 significantly promoted viral replication. Expression of CCL3L1 and TNF-*α* in the NC siRNA- and LGP2 siRNA-transfected cells infected by FMDV was detected by qPCR at 18 hpi. It showed that the expression of CCL3L1 and TNF-*α* was significantly increased after knockdown of LGP2 in the virus-infected cells (Figure 4c).

The CRISPR–Cas9 system was used to establish an LGP2 knockout PK-15 cell line. Knockout of LGP2 in the cell lines was determined by DNA sequencing analysis. The sequencing results indicated that two-nucleotide deletion was introduced into the first exon of one allele gene of LGP2 (LGP2-KO-1), and a four-nucleotide deletion was introduced into the other allele gene of LGP2 (LGP2-KO-2) (Figure 4d,Supplementary Figure 2). One cell line that included the WT LGP2 genome was also obtained in parallel and used as a control. Western blot analysis was performed and showed that LGP2 was not detected in the knockout cell line (LGP2-KO); however, it was detected in the WT cell line (LGP2-WT) (Figure 4e). LGP2-KO and LGP2-WT cells were infected by equal amounts of FMDV, and viral RNA and viral protein levels were detected at 10 hpi. Knockout of LGP2 significantly enhanced viral replication (Figure 4f). These results confirmed the essential role of LGP2 to suppress FMDV replication in the cells. The expression levels of CCL3L1 and TNF-*α* were also detected in the LGP2-WT or LGP2-KO cells infected by FMDV. As shown in Figure 4g, knockout of LGP2 significantly increased CCL3L1 and TNF-*α* production in infected cells. These results further suggested that LGP2 suppressed the production of the inflammatory mediators and cytokines during FMDV infection.

### FMDV infection downregulated LGP2 protein, and FMDV L^pro^, 3C^pro^ and 2B proteins were responsible for this reduction

**Figure 5:**
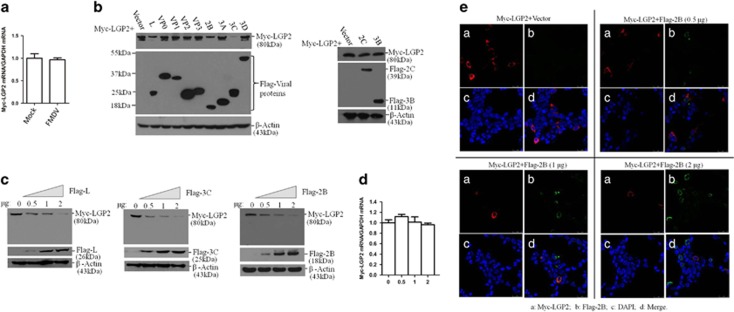
FMDV 2B protein induces reduction of LGP2. (**a**) PK-15 cells were transfected with Myc-LGP2 plasmids for 24 h followed by mock infection or infection with FMDV (0.5 MOI) for 12 h, and Myc-LGP2 mRNA levels were detected by qPCR. (**b**) HEK293T cells were transfected with Myc-LGP2 plasmids (2 μg) along with various plasmids expressing Flag-tagged viral proteins (L^pro^, VP0, VP1, VP2, VP3, 2B, 2C, 3A, 3B, 3C^pro^ or 3D^pol^) or empty Flag vector plasmid (2 μg). Expression of Myc-LGP2 and Flag-tagged viral proteins was detected by western blotting at 36 hpt. (**c**) HEK293T cells were co-transfected with Myc-LGP2 plasmids (2 μg) and increasing amounts of Flag-L, Flag-3C or Flag-2B plasmids (0, 0.5, 1 or 2 *μ*g) for 36 h. Empty vector was used in the transfection process to ensure that the cells received the same amounts of total plasmids. Expression of Myc-LGP2 and Flag-tagged viral proteins was detected by western blotting. (**d**) HEK293T cells were co-transfected with Myc-LGP2 plasmids (2 μg) and increasing amounts of Flag-2B plasmids (0, 0.5, 1 or 2 μg) for 36 h. Empty vector was used in the transfection process to ensure that the cells received the same amounts of total plasmids. Expression of Myc-LGP2 mRNA levels were detected by qPCR. (**e**) HEK293T cells were co-transfected with Myc-LGP2 plasmids (2 μg) and increasing amounts of Flag-2B plasmid (0, 0.5, 1 or 2 μg) for 36 h. Expression of Myc-LGP2 and Flag-2B was detected by immunofluorescence assay. Cells were double-immunostained for Myc-LGP2 (red) and Flag-2B (green); the cellular nuclei were counterstained with DAPI (blue).

To explore whether FMDV inhibited LGP2 expression by disrupting its mRNA, the mRNA levels of Myc-LGP2-transfected cells that were mock-infected or infected by FMDV were detected using qPCR. The results suggested that FMDV infection had no effect on Myc-LGP2 mRNA expression (Figure 5a).

To investigate the viral proteins that were responsible for this reduction, HEK-293T cells were co-transfected with Myc-LGP2 and plasmids expressing various Flag-tagged viral proteins. Expression levels of LGP2 protein was determined at 36 hpt by western blotting. It was observed that the expression of L^pro^, 3C^pro^ and 2B proteins significantly decreased LGP2 protein abundances (Figure 5b). L^pro^-, 3C^pro^- or 2B-induced reduction of LGP2 was further confirmed and compared by performing dose-dependent experiments. It indicated that L^pro^, 3C^pro^ and 2B all play significant role in downregulation of LGP2; and they almost showed approximately similar inductive ability to reduce LGP2 protein levels (Figure 5c). FMDV L^pro^ and 3C^pro^ possess viral proteinase activity that can cleave many host proteins or suppress the expression of several host proteins. LGP2 might be a target for L^pro^ and 3C^pro^. As for 2B-induced reduction of LGP2, it revealed a new mechanism evolved by FMDV to antagonize host antiviral effect. The affection of 2B on Myc-LGP2 transcripts was evaluated, which suggested that 2B did not disrupt the transcripts of Myc-LGP2 (Figure 5d). An indirect immunofluorescence assay was further carried out to reveal 2B-induced decrease of LGP2 protein. It showed that high expression of 2B significantly decreased expression of LGP2 protein, suggesting the inductive ability of 2B to decrease LGP2 in a dose-dependent manner (Figure 5e).

### Essential regions involved in antiviral activity of LGP2 during FMDV replication

**Figure 6:**
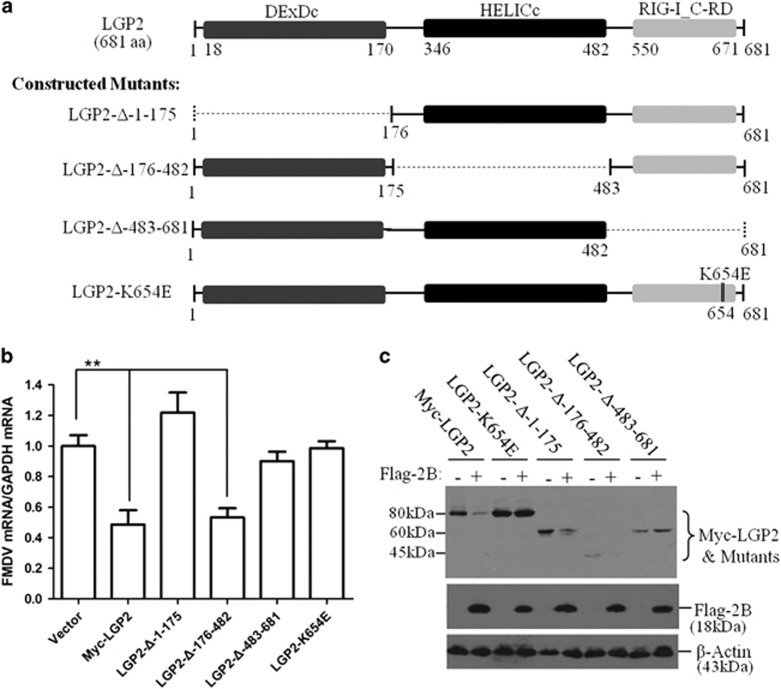
Functional domain of LGP2 to suppress FMDV replication. (**a**) Schematic representation of a series of Myc-tagged LGP2 mutant constructs. (**b**) PK-15 cells were transfected with the empty vector (2 μg), Myc-LGP2 (2 μg) or various LGP2 mutant plasmids (2 μg) for 24 h, and followed by infection with FMDV (0.5 MOI) for 12 h. Viral RNA levels were detected by qPCR. (**c**) HEK293T cells were co-transfected with Myc-LGP2 plasmids (2 μg) or various LGP2 mutant plasmids (2 μg) and Flag-2B plasmid for 36 h. Expression of Myc-LGP2, Myc-tagged LGP2 mutants and Flag-2B proteins was detected by western blotting.

The crucial regions of LGP2 that are associated with its antiviral function were further determined by construction of a series of mutants of Myc-LGP2 plasmids (Figure 6a). The LGP2-Δ-1-175 deleted the N-terminal DEAD-like helicase superfamily ATP binding domain (DExDc) of LGP2. The LGP2-Δ-176-482 deleted the helicase superfamily C-term domain associated with DExH/D box proteins (HELICc) of LGP2. The LGP2-Δ-483-681 deleted the C-terminal regulatory domain (CRD) of LGP2. LGP2-K654E was a mutant that was constructed to disrupt RNA recognition by the CRD of LGP2.^27, 28^ The antiviral activity of these mutants against FMDV indicated that deletion of the N-terminal DExDc or the CRD abolished the antiviral activity of LGP2. Deletion of HELICc retained the antiviral role of LGP2. The site mutation of K654E also resulted in the inability of LGP2 to suppress FMDV replication (Figure 6b).

The inductive activity of 2B to decrease the constructed LGP2 mutants was subsequently evaluated. As shown in Figure 6c, the protein levels of WT LGP2, LGP2-Δ-1-175 and LGP2-Δ-176-482 were reduced by 2B. However, 2B failed to induce reduction of LGP2-K654E and LGP2-Δ-483-681 mutant protein levels. This implied that lysine 654 (K654) and the CRD of LGP2 were the potential targets for 2B to induce reduction of LGP2.

### C-terminal region of 2B was essential for inducing reduction of LGP2 and upregulation of inflammatory cytokines production

**Figure 7:**
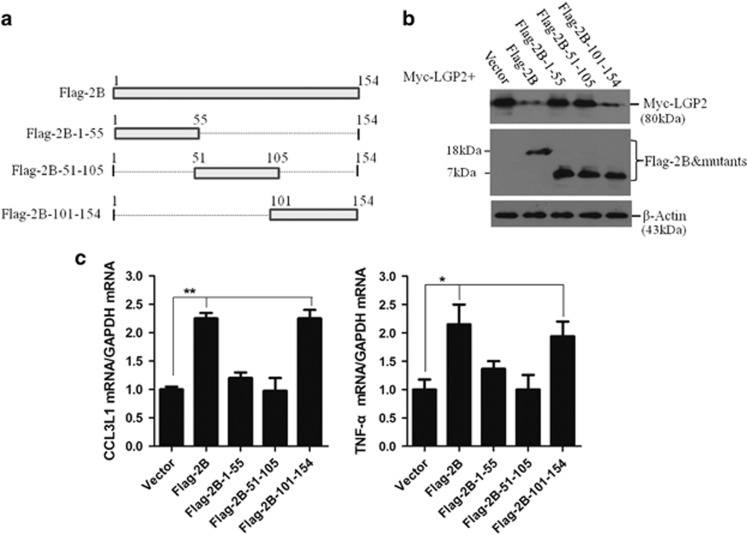
Functional region of 2B responsible for inducing reduction of LGP2. (**a**) Schematics of a series of Flag-tagged 2B mutant constructs. (**b**) HEK293T cells were co-transfected with Myc-LGP2 plasmid (2 μg) and Flag-2B or Flag-tagged 2B mutant plasmids (2 μg) for 36 h. Expression of Myc-LGP2, Flag-2B and Flag-tagged 2B mutant proteins were detected by western blotting. (**c**) PK-15 cells were transfected with the plasmids expressing Flag-2B or truncated mutants, and the cells were infected with equal amounts of FMDV at 12 hpt. Expression of CCL3L1 and TNF-*α* was detected by qPCR at 18 hpi.

To explore the functional region responsible for the activity of 2B to decrease LGP2, a series of truncation mutants of Flag-2B plasmids that were preserved in our lab were used in this study (Figure 7a). As shown in Figure 7b, the truncated mutant that included the 101–154 region (Flag-2B-101–154) induced reduction of Myc-LGP2; however, the truncated mutants containing the 1–55 or 51–105 regions failed to trigger the reduction of Myc-LGP2. This suggested that the C-terminal 101–154 region was essential for reducing Myc-LGP2. The affection of Flag-2B or truncated mutants on FMDV-induced inflammatory response was also investigated. It showed that the expression of CCL3L1 and TNF-*α* was significantly enhanced by tranfection of Flag-2B and Flag-2B-101-154 plasmids in the virus-infected cells (Figure 7c).

### 2B interacted with LGP2 and 2B-induced reduction of LGP2 was independent of proteasomes, lysosomes and caspases pathways or cleavage of eIF4G

**Figure 8:**
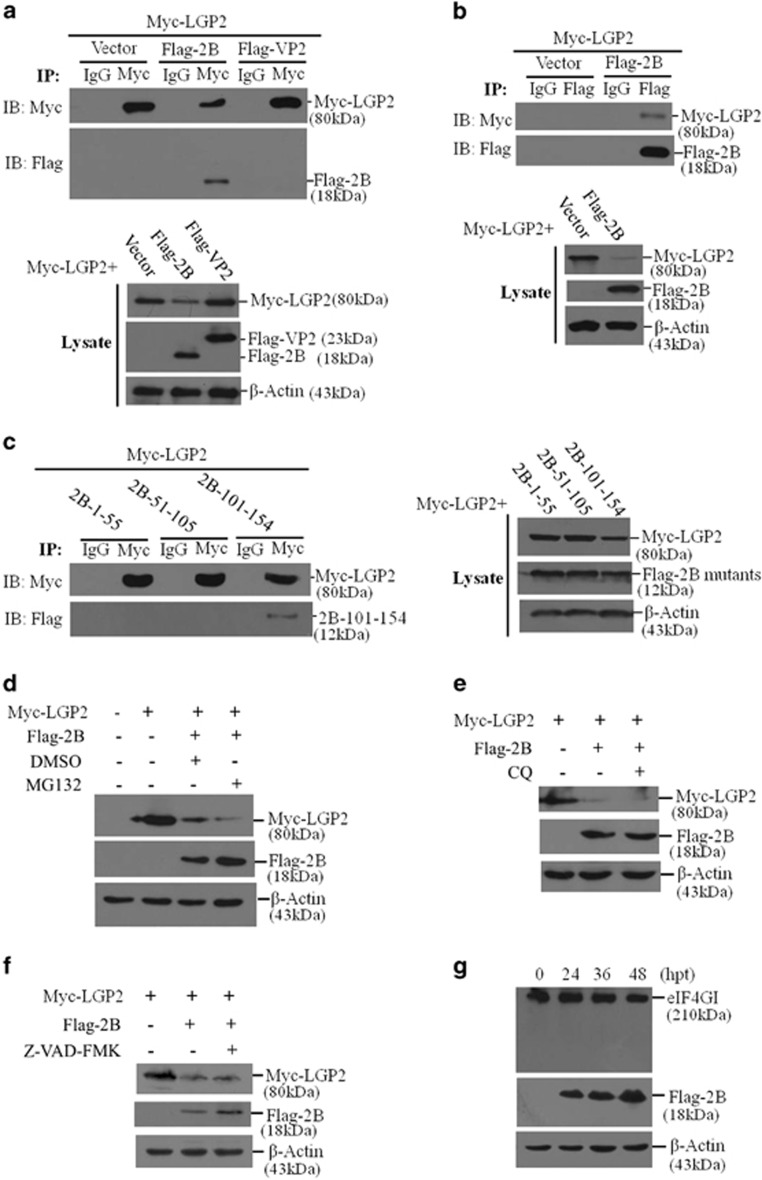
Exploration of the mechanism of 2B-induced reduction of LGP2. (**a**) HEK-293T cells were co-transfected with Myc-LGP2 and empty vector, Flag-2B or Flag-VP2 plasmids for 36 h. Cell lysates were immunoprecipitated with mouse anti-Myc or mouse normal IgG antibody and subjected to western blotting (upper panel). Whole-cell lysates were also directly detected by western blotting to confirm expression of target proteins (lower panel). (**b**) HEK-293T cells were co-transfected with Myc-LGP2 and empty vector or Flag-2B plasmids for 36 h. The cell lysates were immunoprecipitated with rabbit anti-Flag or rabbit normal IgG antibody and subjected to western blotting (upper panel). Whole-cell lysates were also directly detected by western blotting to confirm expression of target proteins (lower panel). (**c**) HEK-293T cells were co-transfected with Myc-LGP2 and Flag-2B mutant plasmids for 36 h. Cell lysates were immunoprecipitated with mouse anti-Myc or mouse normal IgG antibody and subjected to western blotting (left panel). Whole-cell lysates were also directly detected by western blotting to confirm the expression of the target proteins (right panel). (**d–f**) HEK293T cells were co-transfected with Myc-LGP2 plasmids (2 μg) and Flag-2B (2 μg) in the absence or presence of (**d**) 10 μM of MG132, (**e**) 50 μM of chloroquine or (**f**) 50 μM of Z-VAD-FMK for 24 h. Expression of Myc-LGP2 and Flag-2B was detected by western blotting. (**g**) PK-15 cells were transfected with Flag-2B plasmid (2 μg) for 0, 24, 36 or 48 h. Protein levels of eIF4GI were determined by western blotting.

We investigated whether there was a direct interaction between 2B and LGP2. VP2 protein that showed no effect on LGP2 protein expression (Figure 5) was used as a control. As shown in Figure 8a, 2B interacted with LGP2, whereas VP2 did not interact with LGP2. A reverse immunoprecipitation was further performed using anti-Flag antibody to confirm the interaction. Flag-2B protein also precipitated Myc-LGP2 protein (Figure 8b). These results suggested a direct interaction between 2B and LGP2. Flag-2B-1–55 and Flag-2B-51–105 did not induce a reduction of LGP2, and the interaction between the three constructed mutants and LGP2 was further determined. Flag-2B-1–55 and Flag-2B-51–105 did not interact with LGP2 (Figure 8c); however, Flag-2B-101–154 interacted with LGP2. We also evaluated the interaction of 2BC with LGP2, which suggested that 2BC interacted with LGP2 and suppressed LGP2 expression that resembled 2B-mediated inhibitive effect against LGP2 (Supplementary Figure 3a). Our previous study determines that 2B interacts with RIG-I and represses RIG-I expression.^29^ LGP2 was shown to interact with 2B, therefore, we investigated whether LGP2, 2B and RIG-I could form a complex. It was determined that Myc-LGP2 did not precipitate HA-RIG-I in the absence or presence of 2B protein, suggesting that 2B, RIG-I and LGP2 did not form a complex (Supplementary Figure 3b).

To further explore the pathways involved in 2B-induced reduction of LGP2, the proteasome inhibitor MG132, the lysosomal inhibitor chloroquine and the caspase inhibitor Z-VAD-FMK were used in this study. The results indicated that the proteasome inhibitor MG132 showed no inhibitory effect on 2B-induced reduction of LGP2 (Figure 8d). Chloroquine and Z-VAD-FMK also had no effect on the reduction of LGP2 induced by 2B (Figures 8e and f). FMDV infection induces cleavage of eIF4GI to suppress host protein synthesis. We also investigated the possibility of inducing the cleavage of eIF4GI by 2B to inhibit LGP2 synthesis. The kinetics of eIF4GI protein levels were examined in Flag-2B-transfected PK-15 cells. Expression of 2B had no effect on eIF4GI protein levels (Figure 8g). These results indicated that reduction of LGP2 induced by 2B did not depend on the proteasomes, lysosomes or caspase pathways, and was independent of cleavage of eIF4G.

## Discussion

LGP2 is an important RNA helicase that is involved in immunity against various viruses,^22, 26^ but the function of LGP2 in virus recognition and signalling remains controversial.^16, 30^ LGP2 is speculated to have different roles in different viral infections.^19, 24, 31, 32^ It is thought that LGP2 is important for the recognition of picornaviruses, including EMCV and mengovirus.^24, 33^ LGP2 is required for IFN response in EMCV-infected cells.^34^ IAV infection induces sustained upregulation of LGP2 and LGP2 performs an antiviral role against IAV infection.^26, 32^ In this study, we showed for the first time the antiviral activity of LGP2 against FMDV.

Transgenic mice overexpressing LGP2 displayed a significantly decreased inflammatory response and improved survival ratio compared with WT mice after IAV infection.^26^ Severe inflammation is associated with FMDV-induced diseases.^35, 36, 37, 38^ We therefore investigated the impact of LGP2 overexpression on the inflammatory response to FMDV; and we found a similar suppressive effect on the inflammatory response induced by overexpression of LGP2 in FMDV-infected cells. This is in fair agreement with data obtained from IAV-infected transgenic mice overexpressing LGP2.^26^ TNF-*α* and IL-6 are significant proinflammatory cytokines in virus-infected cells.^39^ This suggests that LGP2 could inhibit inflammatory response by inhibiting TNF-*α* and IL-6 production. Sustained exposure to high levels of IL-4 can induce a striking phenotype of tissue infiltration and result in severe inflammatory disease.^40^ Overexpression of LGP2 significantly suppressed IL-4 expression in FMDV-infected cells, which may have partially contributed to the decreased viral replication. IL-4 can increase IL-10 production;^40, 41^ In LGP2-overexpressing cells, the level of IL-10 was lower than in vector-transfected cells. The reduction in IL-4 may have been responsible for it. IL-10 production at the early stage of FMDV correlates with the development of persistent infection in cattle.^42^ Downregulation of IL-10 may promote virus clearance in the infected cells. Levels of GM-CSF have been demonstrated to increase during inflammatory responses.^43^ A significant decrease of GM-CSF was observed in LGP2 overexpressing cells, which also suggested the suppressive effect of LGP2 on inflammatory response. IL1R*α* blocks proinflammatory cytokine IL-1-mediated inflammation.^44^ PECAM-1 is also implicated in the regulation of inflammatory responses. PECAM-1 suppresses proinflammatory cytokine production, showing anti-inflammatory activity in C57BL/6 mice.^45, 46^ Upregulation of IL1R*α* and PECAM-1 in LGP2 overexpressing cells might also inhibit the virus-induced inflammatory response. This revealed the essential role of LGP2 in suppressing the inflammatory response in FMDV-infected cells. Previous studies have hypothesized LGP2 as a concentration-dependent biphasic switch to reconcile dual LGP2 functions in antiviral signalling.^25^ Low levels of LGP2 are believed to be synergistic with MDA5. The sensing of FMDV is solely mediated by MDA5 but not RIG-I in PK-15 cells.^47^ LGP2 is present at low levels in the PK-15 cells (Figure 1). It implies the potential role of LGP2 in regulation of MDA5 signalling during FMDV infection. Therefore, FMDV infection induces the decrease of LGP2 to promote virus replication. The high expression of LGP2 is deemed to inhibit MDA5 signalling activity to drive IFN-*β* expression back towards baseline.^21, 25, 48^ Overexpression of LGP2 suppressed the inflammatory response in FMDV-infected cells; this possibly was due to this inhibitive effect of LGP2. LGP2 has also been reported to be associated with radioresistance in numerous diverse cancer cell lines.^49, 50^ Our results could provide an insight to investigate the connection between LGP2 and inflammatory responses in the tumorigenesis. These findings demonstrate the significance of further studies to fully understand the mechanisms of LGP2 function in various contexts.

The paramyxovirus V protein can target the LGP2 helicase domain, disrupting its ATP hydrolysis activity, thus benefiting viral replication.^51^ L^pro^ and 3C^pro^ are widely known as viral proteinases in picornaviruses.^52^ FMDV L^pro^ and 3C^pro^ are responsible for several of the viral polyprotein cleavages; and various host proteins can also be cleaved by L^pro^ and 3C^pro^.^4^ 2B protein did not include any potential proteolytic domains.^29^ 2B protein can induce re-arrangement of host cell membranes and disruption of the cellular secretory pathway, which may promote apoptosis of host cell.^53, 54^ FMDV 2B has viroporin activity that may also affect apoptosis.^55^ However, our previous study showed that FMDV 2B did not induce apoptosis in PK-15 cells.^29^ In this study, we also demonstrated that 2B had no effect on eIF4G-mediated host protein synthesis; and 2B-induced reduction of LGP2 was independent of the proteasome, lysosome or caspase pathway. It indicates that 2B may repress LGP2 expression by some complicated mechanisms.

The N-terminal DExDc and CRD domains were essential for LGP2-mediated anti-FMDV activity (Figure 6). The N-terminal DExDc is associated with the ATP-binding and ATPase activity of LGP2, and CRD is significantly involved in RNA recognition and binding activity.^48, 56^ This implies that the ATP binding, ATPase and RNA recognition activities of LGP2 are involved in its antiviral response. The porcine LGP2-K654E is a mutant similar to human LGP2 K651E that completely disrupts RNA recognition by the isolated CRD.^48^ LGP2-K654E lost its antiviral activity against FMDV, suggesting that RNA recognition is significant for LGP2-mediated antiviral activity during FMDV infection. HELICc is mainly associated with the unwinding dsRNA activity (helicase activity). The HELICc domain of the helicase DDX41, which belongs to the DEXDc family of helicases, has a suppressive effect on IFN-*β* promoter activity. Overexpression of DDX41 lacking the HELICc domain results in enhanced activation of IFN-*β* promoter than overexpression of full-length DDX41.^57^ However, in this study, we observed that the HELICc domain of LGP2 was not essential for antiviral activity during FMDV infection. This implies that the HELICc domain in different helicase may perform different roles.

Our previous study indicated that 2B interacts with RIG-I and suppresses RIG-I expression.^29^ In this study we did not observe a complex interaction of RIG-I, LGP2 and 2B. This indicated that 2B may interact with LGP2 or RIG-I through a similar domain to perform its antagonistic function, and some other proteins might be involved in this process. The mechanisms of 2B-induced downregulation of LGP2 or RIG-I remain unclear, we deem that 2B may recruit several host proteinase proteins to form a complex to induce the proteolysis of RIG-I and LGP2. Further studies should be performed to explore several proteins with proteinase activity that interact with 2B.

In conclusion, the present study demonstrated the antiviral role of LGP2 by repressing the inflammatory response during FMDV infection. It also showed a novel mechanism by which FMDV 2B has evolved to induce a reduction of LGP2 and counteract LGP2-induced antiviral effect.

## Materials and Methods

### Cells

Baby hamster kidney-21 (BHK-21), pig kidney epithelial cells (PK-15) cells and human embryonic kidney 293T (HEK293T) cells were purchased from the Cell Bank of Type Culture Collection of Chinese Academy of Sciences (Shanghai, China). All media and reagents were purchased from Life Technologies (Carlsbad, CA, USA). All the cells were maintained in Dulbecco's modified Eagle medium (DMEM) (Gibco) supplemented with 10% fetal bovine serum (FBS) (Gibco, Grand Island, NY, USA) and maintained at 37 °C (5% CO_2_).

### Virus, viral infection and TCID_50_ assay

FMDV type O strain O/BY/CHA/2010 was isolated in China in 2010 and conserved by National Foot and Mouth Diseases Reference Laboratory, Lanzhou Veterinary Research Institute, Chinese Academy of Agricultural Sciences. O/BY/CHA/2010 strain was used for all the viral challenges in this study. The virus was propagated in BHK-21 cells. Viral infection experiments were carried out as described below. Cell cultures were washed with phosphate-buffered saline (PBS) for three times, and the virus was incubated for 1 h in serum-free medium, washed with PBS. The infected cells were then maintained in fresh medium supplemented with 1% FBS. The total proteins of FMDV-infected cells were extracted using RIPA lysis buffer (Beyotime, China) at different hours post-infection (hpi). The 50% tissue culture infective dose (TCID_50_) values were determined using the Reed-Muench method. BHK cells were seeded on 96-well plates (5 × 10^4^ cells each well) and the monolayer cells were washed with PBS and overlaid with serially diluted virus-containing samples. After 1 h adsorption, the supernatant was removed and the cells were maintained in fresh medium supplemented with 1% FBS for 72 h at 37 °C. The viral cytopathic effect were observed and recorded, the TCID_50_ was calculated as described previously.^58^

### Antibodies and plasmids

Mouse anti-Myc monoclonal antibody, rabbit anti-Flag monoclonal antibody and mouse anti-*β*-actin monoclonal antibody were purchased from Santa Cruz Biotechnology (Santa Cruz, CA, USA). Mouse anti-Flag monoclonal antibody was purchased from Sigma–Aldrich (St. Louis, MO, USA). Rabbit anti-LGP2 polyclonal antibody and rabbit anti-eukaryotic translation initiation factor 4 gamma (eIF4G) polyclonal antibody were purchased from Abcam(Cambridge, MA, USA). Anti-VP1 polyclonal antibody was prepared by our laboratory (unpublished data). The full-length cDNA of LGP2 was amplified from PK-15 cells and cloned into pcDNA^TM^3.1/myc-His(-)A vector (Invitrogen, Carlsbad, CA, USA) to generate the Myc-tagged expressing plasmid (Myc-LGP2). Various Flag-tagged viral protein expressing plasmids were constructed by our lab previously as described.^3^ A series of Myc-tagged LGP2 mutant constructs were generated by site-directed mutagenesis PCR.^59^ All the generated expressing plasmids were analysed and verified by DNA sequencing.

### RNA extraction and quantitative real-time PCR (qPCR)

Total RNAs were extracted using the TRIzol^®^ Reagent (Invitrogen) according to the manufacturer's protocol. Two-micrograms (μg) of RNAs were used as the template, and the M-MLV reverse transcriptase (Promega, Madison, WI, USA) and random hexamer primers (TaKaRa, Dalian, China) were used to synthesize cDNAs. The yield cDNAs were detected and quantified by quantitative real-time PCR (qPCR) using SYBR Premix *Ex* Taq reagents (TaKaRa) and the Mx3005P QPCR System (Agilent Technologies, Palo Alto, CA, USA). Relative abundance of mRNA was calculated using the comparative cycle threshold (CT) (2^−ΔΔCT^) method.^60^ The glyceraldehyde-3-phosphate dehydrogenase (GAPDH) gene was used as the endogenous control. All the qPCR experiments were performed three times. The data represent results from one of the triplicate experiments. *P<0.05 considered significant, **P<0.01 considered highly significant. The qPCR primers used in this study were shown in Table 1.

### Transfection of DNA plasmids and siRNAs

All the DNA plasmids were transfected into the cells using the transfection reagent Lipofectamine™ 2000 (Invitrogen) according to the manufacturer's instruction. As for the transfection of siRNAs, the PK-15 cells were seeded on six-well plates (5 × 10^5^ cells each well) and grown at 37 °C to a confluence of 60–70%, washed with PBS, and incubated with transfection complex containing 150 nM siRNA and 7.5 μL Lipofectamine™ 2000 in Opti-MEM I Reduced Serum Medium (Gibco) for 6 h. The supernatant was removed and the cells were maintained in DMEM supplemented with 10% FBS. All the siRNAs were purchased from Genepharma Company (China). Non-targeting siRNA (NC siRNA) was used as a negative control for LGP2 siRNA. The target sequence for porcine LGP2 was 5′-GGGACCAGCAAGAAGTGA-3′.

### Cytokine array profiling

Expression of 20 different cytokines was detected and analysed using antibody-based porcine cytokine array kits (RayBiotech, Inc., Norcross GA, USA). The PK-15 cells (1 × 10^6^) were seeded in 60 mm culture dishes, and 4 *μ*g of the vector or Myc-LGP2 plasmids were transfected into the cells and infected with FMDV at a multiplicity of infection (MOI) of 0.5 at 24 h hpt. The cytokines expression levels in the cell lysates were detected at 18 hpi according to the manufacturer's protocol.

### Generation of the LGP2 knockout PK-15 cell line

The single guide RNA (sgRNA) sequence targeting porcine LGP2 was designed using the online clustered regularly interspaced short palindromic repeats (CRISPR) design tool (http://crispr.mit.edu/). The designed sequence was 5′-GGAGGTGATCATGCCCGCTC-3′. The synthesized sgRNA fragment was annealed and cloned into pX330 plasmid expressing Cas9 (Addgene plasmid 42230). PK-15 cells were grown on 12-well plates (3 × 10^5^ cells each well) and cultured to a confluence of approximately 60**–**70% and followed by transfection with 2 *μ*g of the constructed plasmid. The genomic DNA was extracted using DNeasy Blood and Tissue Kit (QIAGEN, Valencia, CA, USA) according to the manufacturer's protocol at 72 hpt. The LGP2 genomic DNA surrounding the designed CRISPR target site was analysed by PCR detection using the check primers (forward: 5′-TGTGGTCCTTAGTCCTCTGCC-3′ reverse: 5′-TCACCGTTGAGCTCCACGT-3′). The amplified fragments were purified and re-annealed as described for sgRNA annealing. The genome editing of LGP2 was determined by T7 Endonuclease I (New England Biolabs, Ipswich, MA, USA). The re-annealed fragments digested by T7 Endonuclease I were separated and analysed on a 1.5% agarose gel. After confirmation of the available editing activity of the synthesized sgRNA, the transfected cells were cultured by limiting dilution. The LGP2 knockout single-clone cell line was separated and obtained by the limiting dilution method in 96-well plates (0.5 cell each well). The genomic DNA of the cells cultured from a single-cell clone was analysed by PCR using the check primers, and the amplified fragments were purified and ligated into pMD-18T vectors. The frame-shifting mutation of both alleles of the established single clone cell line was identified by sequencing analysis. Six plasmids from each of the single-clone cell line samples were sequenced and analysed. Western blot analysis was performed to confirm the knockout of LGP2 in the established cell line. The wild-type (WT) PK-15 cell line was established in parallel and used as a control.

### Co-immunoprecipitation assay and western blotting

Cells were seeded in 10-cm dishes and cultured to a confluence of 60–70% and followed by transfection of various indicated plasmids. The collected cells were lysed using the lysis buffer (20 mM Tris-HCl (pH 7.4–7.5), 150 mM NaCl, 1 mM EDTA, 1%Nonidet *P*-40, 10 mg/ml aprotinin, 10 mg/ml leupeptin, and 1 mM phenylmethylsulfonyl fluoride) as described previously.^3^ Anti-Myc or anti-Flag antibodies were used to immunoprecipitate the interacted proteins by 50% (v/v) slurry of GammaBind G Plus-Sepharose (GE Health Care Life Sciences, Piscataway, NJ, USA) overnight at 4 °C. The precipitates were subjected to western blotting. For western blotting, the protein-containing supernatants were fractionated by 10% SDS-PAGE and transferred onto Immobilon-P membranes (Millipore, Bedford, MA, USA). Membranes were blocked with 5% skimmed milk in TBST. Appropriate antibodies were incubated to generate antibody–antigen complexes. The antibody–antigen complexes were then visualized by enhanced chemiluminescence detection reagents (Thermo Fisher Scientific Inc., Rockford, IL, USA).

### Indirect immunofluorescence microscopy

The cells were seeded in Nunc™ glass bottom dishes and cultured to a confluence of approximately 60–70%, the cells were fixed at 24 hpt with acetone/methanol mixture (1:1) for 10 min at −20 °C. 5% normal goat serum in PBS was used as the blocking buffer; the fixed cells were washed with PBS and blocked for 1 h at 37 °C. The anti-Myc and anti-Flag primary antibodies were subsequently incubated overnight at 4 °C. Cells were washed with PBS for five times at room temperature (10 min each time). The fluorochrome-conjugated secondary antibodies were incubated on the cells in dark for 1 h at room temperature, washed with Tris-buffered saline (pH 7.6) for three times at room temperature (10 min each time). The cells were incubated with 4′,6-diamidino-2-phenylindole (DAPI, Roche, Diagnostics, Mannheim, Germany) for 10 min at room temperature to stain the nuclei. The stained cells and fluorescence were visualized using a Nikon eclipse 80i fluorescence microscope with appropriate settings. The microscopy images were processed using NIS Elements F 2.30 software.

### Statistical analysis

All the quantified results in this study were present as mean values±s.e. of three independent experiments. The Student's *t*-test was used to determine statistical significance. *P<0.05 was considered significant, **P<0.01 was considered highly significant.^61^

## Figures and Tables

**Table 1:** The qPCR primers used in this study

*Gene*	*Primers (5′→3′)*
LGP2	Forward: CAGCCCTGCAAACAGTACGAC
	Reverse: CACTCCAGTTTCGGGTTCTC
	
GAPDH	Forward: ACATGGCCTCCAAGGAGTAAGA
	Reverse: GATCGAGTTGGGGCTGTGACT
	
FMDV	Forward: CACTGGTGACAGGCTAAGG
	Reverse: CCCTTCTCAGATTCCGAGT
	
TNF-*α*	Forward: CGCCCACGTTGTAGCCAATGT
	Reverse: CAGATAGTCGGGCAGGTTGATCTC
	
CCL3L1	Forward: TCTCGCCATCCTCCTCTG
	Reverse: TGGCTGCTGGTCTCAAAATA
	
IL1*α*	Forward: AGAATCTCAGAAACCCGACTGTTT
	Reverse: TTCAGCAACACGGGTTCGT
	
IL13	Forward: AAGTGGCCCAGTTCGTAAAAGA
	Reverse: ACCCGTGGCGAAAAATCA
	
ILra	Forward: CCTTCATCCGCTCCGACA
	Reverse: GTGACCTTGACGGCTGCTTT
	
PECAM-1	Forward: AAAGGGCACGGAGACAG
	Reverse: GGGCAGGTTCATAAATAAGT
