# Supplementary material for: Foot-and-mouth disease virus infection inhibits LGP2 protein expression to exaggerate inflammatory response and promote viral replication
Source: Cell Death Dis. 2017 Apr 13;8(4):e2747–. doi: 10.1038/cddis.2017.170 (PMC5477588; doi:10.1038/cddis.2017.170)
Supplement: Supplementary Table 1 [file cddis2017170x1.pdf]

Supplementary Information

Foot-and-mouth disease virus infection inhibits LGP2 protein expression to exaggerate inflammatory response and promote viral replication

Zixiang Zhu, Chuntian Li, Xiaoli Du, Guoqing Wang, Weijun Cao, Fan Yang, Huanhuan Feng, Xiangle Zhang, Zhengwang Shi, Huanan Liu, Hong Tian, Dan Li, Keshan Zhang, Xiangtao Liu, and Haixue Zheng*

*** Correspondence:** Lanzhou Veterinary Research Institute, Chinese Academy of Agricultural Sciences, No. 1, Xujiaping Road, Lanzhou, 730046, PR China. E-mail: haixuezheng@163.com

Supplemental Figures

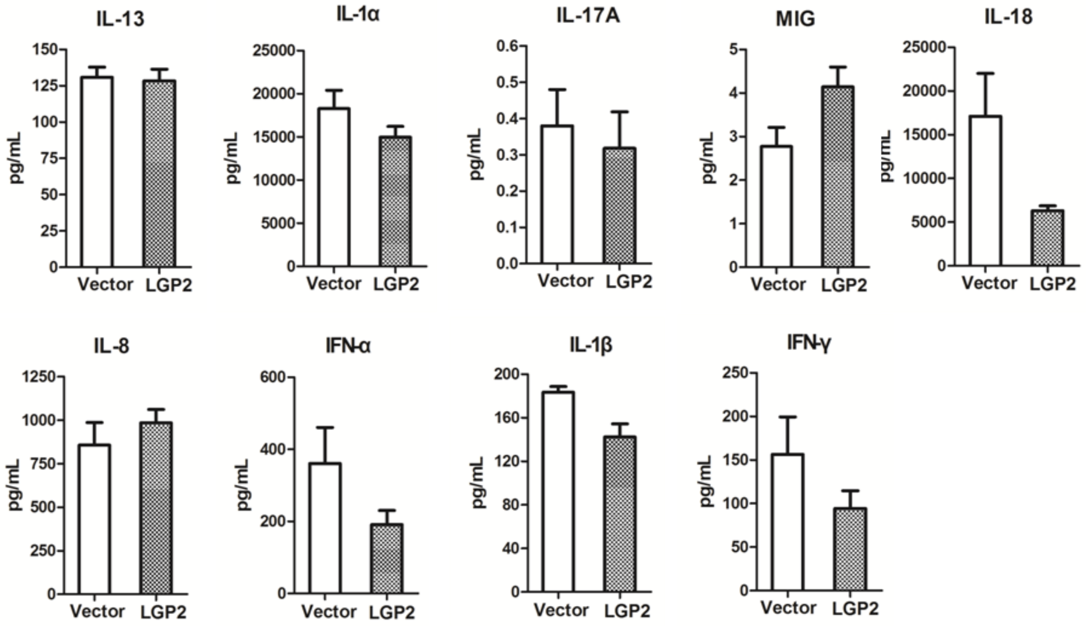

Supplemental Figure 1. Cytokines expression levels that showed no significant changes in LGP2 overexpressing cells compared with vector-transfected cells after FMDV infection.

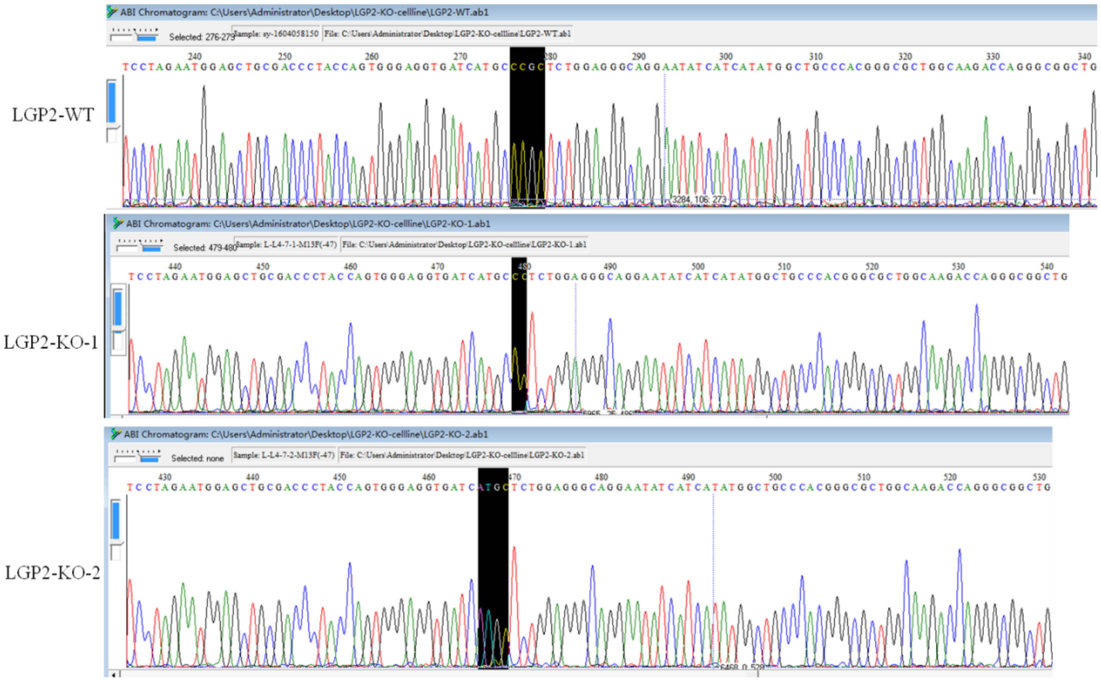

Supplemental Figure 2. Analysis of the genome editing by Sanger sequencing of the PCR amplicon from the genome of LGP2-WT and LGP2-KO cell lines.

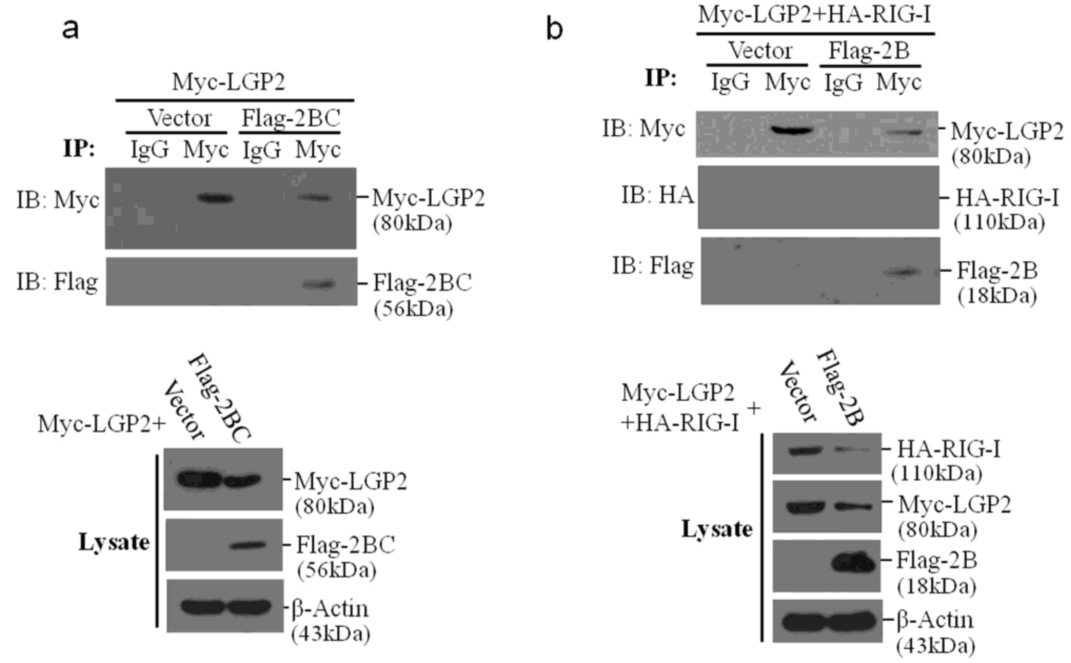

Supplemental Figure 3. Investigation of the interaction of 2BC or RIG-I with LGP2. **(a)** 2BC interacts with LGP2 and induces reduction of LGP2. HEK-293T cells were co-transfected with the Myc-LGP2 and empty vector or Flag-2B plasmids for 36 h. Cell lysates were immunoprecipitated with mouse anti-Myc or mouse normal IgG antibody and subjected to western blotting (upper panel). Whole-cell lysates were also directly detected by western blotting to confirm expression of target proteins (lower panel). **(b)** RIG-I, LGP2 and 2B do not form a interaction complex. HEK-293T cells were co-transfected with the Myc-LGP2 and HA-RIG-I expresssing plasmids and empty vector or Flag-2B plasmids for 36 h. Cell lysates were immunoprecipitated with mouse anti-Myc or mouse normal IgG antibody and subjected to western blotting (upper panel). Whole-cell lysates were also directly detected by western blotting to confirm expression of target proteins (lower panel).
